# Factors Associated With the Utilization of Emergency Contraceptives by Female College Students in Rural Ghana: A Cross‐Sectional Study

**DOI:** 10.1002/hsr2.70575

**Published:** 2025-03-18

**Authors:** Muhusin Alhassan, Yaa Nyarko Adjeso, Clement Tiimim Yanbom, Samuel Kwame Sopuruchi Agomuo

**Affiliations:** ^1^ Midwifery Training College Tumu Upper West Region Ghana; ^2^ Department of Population and Reproductive Health School of Public Health, University for Development Studies Tamale Northen Region Ghana; ^3^ Ghana Health Service, Sissala West District Health Directorate Upper West Region Ghana; ^4^ Department of Molecular Medicine School of Medicine and Dentistry, Kwame Nkrumah University of Science and Technology Kumasi Ashanti region Ghana

**Keywords:** attitude, Emergency contraceptives, female college students, knowledge, practices

## Abstract

**Background and Aims:**

Emergency contraception is a simple and safe method of preventing unwanted pregnancy following an unprotected or nonconsensual sexual encounter. University students have a history of engaging in risky sexual behaviors, leading to unintended pregnancies among female undergraduates. This study assessed the utilization of emergency contraceptives (EC) among female college students in rural Ghana.

**Methods:**

This quantitative cross‐sectional study was conducted among 310 female college students from two institutions – Midwifery Training College and the Tumu College of Education – located in the Sissala East Municipality of the Upper West region of Ghana. A structured questionnaire consisting of four sections was utilized as the data collection instrument. The univariable and multivariable logistic regression was performed to determine the sociodemographic factors and knowledge level associated with emergency contraceptive utilization. Statistical analysis was performed utilizing SPSS v. 25.0 and STATA v. 14.0.

**Results:**

80.2% of students had heard of EC with the health worker (41.6%) and colleagues/friends (30.8%) being the most common sources of information. Overall, the majority of participants had good knowledge (78.2%) and good attitudes (77.8%) regarding EC practice. 52% had used EC before, with more than half (69.5%) utilizing levonorgestrel‐only pill the most. Good Knowledge (aOR: 4.46 [95%CI: 2.28‐8.72]; *p* < 0.001) and being in the Midwifery College (aOR: 1.96 [95%CI: 1.14‐3.38]; *p* = 0.015) were significantly associated with EC utilization.

**Conclusion:**

Despite the majority of participants having heard of EC, most were not utilizing them appropriately due to a lack of detailed information and misconceptions. Health policymakers, heads of tertiary institutions, and educators should prioritize improving the educational curriculum and strengthening advocacy efforts in rural areas to address misconceptions and promote the benefits of EC, ultimately leading to better EC utilization and a reduction in unwanted pregnancies.

## 1 Introduction

Modern contraceptive utilization continues to be the single most important targeted public health intervention and a cost‐effective method to prevent unwanted pregnancies, reduce maternal mortalities, and control rapid population growth [1]. The World Health Organization (WHO) reported that, globally, approximately 210 million pregnancies occur annually out of which 38% are unintended and 22% end in abortions [2]. In sub‐Saharan Africa, 30–43% of unplanned pregnancies result in induced abortions, where two‐thirds of the abortions are classified as unsafe [2]. Reports from WHO have shown that about 5.5 million women in Africa have unsafe abortions, in which 36,000 of them die from abortion related complications and more than a million encounter short and long‐term morbidity and disability [3].

Emergency contraceptives (EC) are contraceptive methods used after unprotected sexual intercourse, sexual assault, missing regular family planning, or failure to use a method [3]. Despite the importance of EC in the reduction of mortality and morbidity, usage or coverage remains low for both developing and underdeveloped nations [4]. Many factors influence the uptake of EC, ranging from cost to availability [5]. For instance, literature reveals that factors associated with EC use include age, educational level, knowledge, culture, religion, sexual activity, previous use of regular contraceptives, and marital status [6, 7, 8].

Ghana has a high unmet need for contraceptive usage with the current usage of any contraceptive method among all women is 23 percent [9]. Moreover, analysis of data from the Municipal Health Directorate annual report revealed an increasing trend of abortions with majority between the ages of 20‐24 years [10]. College‐aged students, known for their sexually active lifestyle, are at elevated risk of unplanned pregnancies. This is largely attributed to sporadic premarital sexual activity, a behavior that could be mitigated through the use of emergency contraception (EC) [11]. Findings from previous studies have proven that if there is access and proper utilization of EC, it has a high chance of averting unwanted pregnancy, reducing unsafe abortions and subsequently preventing maternal deaths [12]. Knowledge and attitudes regarding EC have been identified to influence its utilization [13, 14]. Although these factors among female students have been assessed in Ghana, available research was conducted in urban settings and do not portray the overall rural picture of EC utilization. This study therefore investigated the utilization of emergency contraceptives among female college students in the Sissala East Municipality, a rural Municipal in the Upper West Region of Ghana.

## 2 Methodology

### 2.1 Study Design

A quantitative cross‐sectional study design was utilized to identify the factors associated with the utilization of emergency contraceptives by female college students.

### 2.2 Study Setting

**Figure 1:**
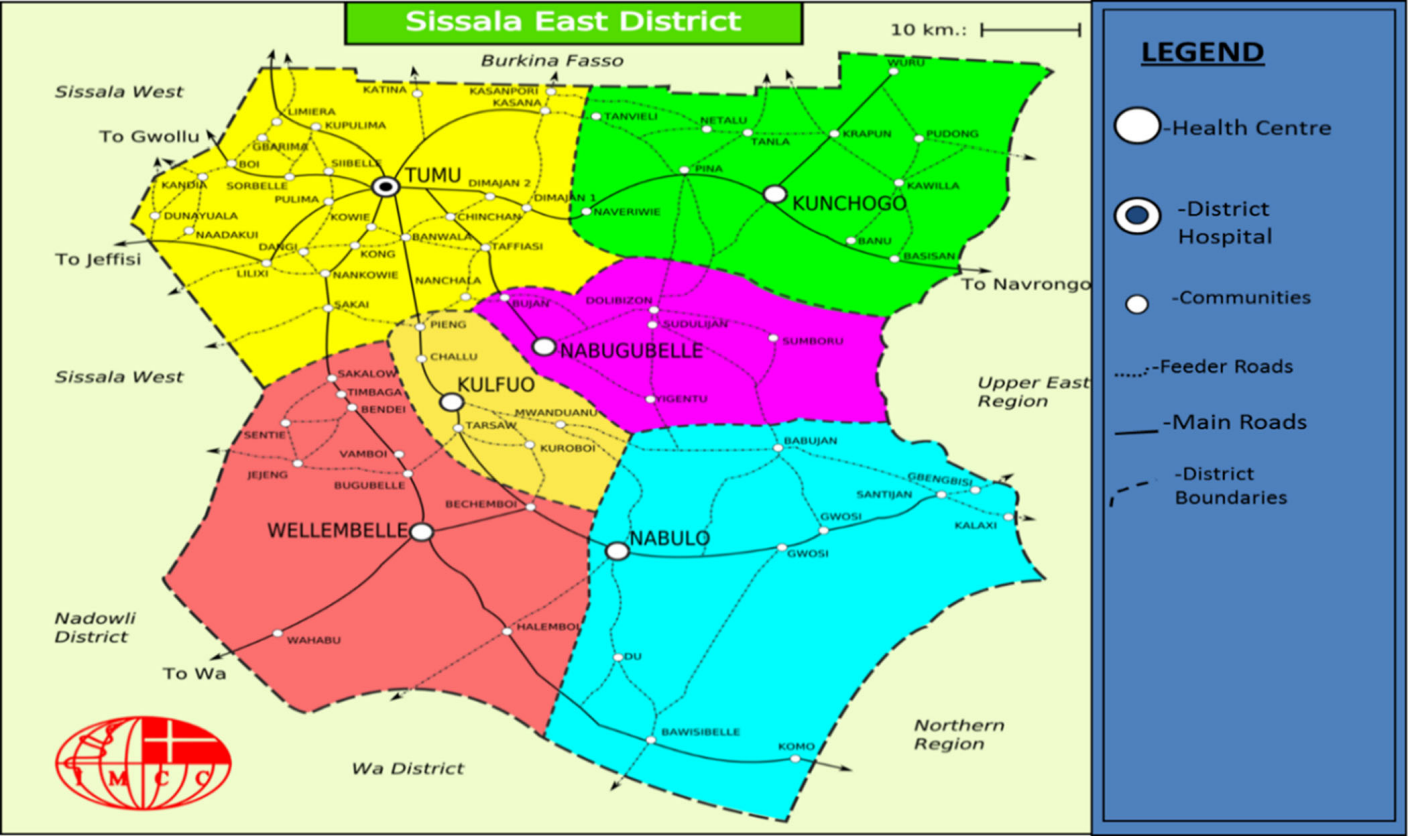
Map of the Sissala East Municipality.

The study was conducted at the Midwifery Training College and the Tumu College of Education, both of which are located in the Sissala East Municipality of the Upper West Region of Ghana (Figure 1). The municipality has a total land size of 4,744 sq. km and falls between longitude 1.300 W and latitude 10.000 N and 11.000 N. Both colleges are located in the municipality's capital, Tumu.

### 2.3 Study Population

A total of 310 female college students who provided written informed consent to participate were included in the study. Male students and women who are not enrolled in a tertiary institution were excluded from participation. Additionally, respondents with incomplete or unclear questionnaire responses were exempted.

### 2.4 Sample Size

The Cochran formula was used to calculate the sample size described below [15]:

$$n=\frac{{\left(Z\frac{\partial }{2}\right)}^{2}p(1–p)}{d^{2}}$$

Where:

n: sample size

p: prevalence of emergency contraceptive utilization in Ghana among female university students = 88.4% (0.884) [16].

d: 5% margin of error

Zα/2 = 1.96 since α = 5% at 95% confidence level

$$n=\frac{\left({1.96)}^{2}\;0.884\;(1-0.884\right)}{0.05^{2}}$$

n = 157.6

A total minimum sample size of 158 was obtained. However, to increase statistical power and account for a 10% nonresponse rate, 310 participants were finally recruited for this study.

### 2.5 Sampling Technique

Stratified and systematic random sampling techniques were utilized to recruit participants for the study. The municipality was stratified into two groups: the College of Education and the Midwifery Training College. The total sample size of 310 participants was proportionally distributed based on the female student population in each institution. Consequently, 190 participants (61%) were allocated to the Midwifery Training College, which had a total female population of 628, while 120 participants (39%) were assigned to the College of Education, which had a total female population of 400. For recruitment, female students were assembled in three classrooms according to their academic level. Participants were selected randomly from each class, with every third individual chosen sequentially until the desired sample size was achieved.

### 2.6 Ethical Considerations

Ethical clearance was sought from the Kwame Nkrumah University of Science and Technology's Ethical Review Committee (CHRPE/AP/038/22). All respondents were asked to provide written informed consent before the commencement of the study. They were informed of their right to withdraw from the study at any time and were assured that their responses would be kept strictly confidential.

### 2.7 Data Collection

A structured questionnaire from the adolescent reproductive health data collection tool by the United Nations Development Programme (UNDP)/United Nations Population Fund (UNFPA) was adapted, modified, and utilized as the main data collection instrument. This data collection tool consisted of closed and open‐ended questions with four sections namely: demographic characteristics, knowledge level, attitudes and utilization of emergency contraceptives. Each participant was educated on the purpose of the study and how the questionnaire was to be filled. A scoring system assigned “1” point for correct answers and “0” for incorrect ones for knowledge and attitude levels. The scores were expressed in percentages that ranged from 0% to 100%. For the scale of interpretation, the cut‐off scores were patterned from the relevant review of literature from similar studies [17, 18]. Scores greater than or equal to 50% were classified good while those less than 50% were poor. Emergency contraceptive utilization was determined based on a binary (yes/no) response to the question, “Have you ever used emergency contraception”

### 2.8 Questionnaire Pretesting

Pretesting of questionnaires was conducted at Saint Clare Vocational School in the Sissala East Municipality with 15 respondents. Reliability of the instrument was ensured by calculating the Cronbach alpha reliability coefficient. After the pilot testing, a Cronbach alpha reliability coefficient value of 0.79 was obtained indicating the reliability of the questionnaire.

### 2.9 Statistical Analysis

Data obtained was entered into SPSS version 25.0 for cleaning and merging, while STATA version 14.0 was used for subsequent analysis. Data was analyzed by employing descriptive and inferential statistics. Frequency, percentages and graphs were used to represent categorical variables while continuous variables were presented as mean ± standard deviation (SD). The Pearson's Chi‐square test was used to identify the relationship between demographic features and the utilization of emergency contraceptives. The univariable and multivariable logistic regression was performed to identify the independent predictors of emergency contraceptive utilization. Multicollinearity among independent variables was assessed using the Variance Inflation Factor (VIF), and model fit was evaluated using the Hosmer‐Lemeshow goodness‐of‐fit test. Statistical significance was determined using a two‐tailed test, with *p*‐values less than 0.05 considered statistically significant.

## 3 Results

### 3.1 Sociodemographic Characteristics of Study Participants

Of the 310 respondents recruited in this study, two‐thirds of respondents (67.9%) were among the ages of 26 to 30 years with a mean age of 23.14 ± 2.97 years. About a third (31.6%) were from the Sissala tribe and just above half of participants (58.9%) were Christian. Additionally, almost two‐thirds (61.3%) of the female college students were from the Midwifery College while 39.7% were from the College of Education. Regarding the year of study, above one‐third (37.9%) were in the third year followed by first year (32.4%) and second year (29.8%) (Table 1).

**Table 1:** Sociodemographic characteristics of study participants.

Variable	Frequency (*n* = 310)	Percentage (%)
Age group (years)		
< 21	50	16.6
21–25	205	67.9
26–30	37	12.3
> 30	10	3.3
Mean age = 23.14 ± 2.97, range 18–34		
Ethnicity		
Sissala	98	31.6
Dagare	68	21.9
Akan	47	15.2
Frafra	25	8.1
Wali	18	5.8
Kasem	18	5.8
Others	36	11.6
Religion		
Christian	182	58.9
Muslim	128	41.1
Institution of training		
College of Education	120	38.7
Midwifery college	190	61.2
Year at college		
Year 1	100	32.4
Year 2	93	29.8
Year 3	117	37.9

*Note:* Data presented as frequency and percentages unless otherwise stated. Age was also presented as mean ± standard deviation.

### 3.2 Awareness of Emergency Contraceptives

The research findings indicated that a vast majority (80.2%) of female college participants had heard of emergency contraceptives before with only 19.8% who have not heard of it. The major sources of information for those who have heard of EC were from health workers (41.6%), colleagues or friends (30.8%), and the media (23.1%). Almost half of the respondents (48.8%) heard about EC over 3 years ago while 28.8% heard of it between 6 months to 3 years ago during the study. Moreover, almost all participants (92.7%) knew measures to prevent pregnancy after unprotected sex with just a few (7.3%) having no idea (Table 2).

**Table 2:** Participant awareness regarding emergency contraceptives.

Variable	Responses	Frequency (n = 310)	Percentage (%)
Awareness	Yes	249	80.2
	No	61	19.8
Source of information	Family/Parents	6	2.5
	Colleagues/Friends	77	30.8
	Media	58	23.1
	Health Worker	104	41.6
	Others	5	2.1
First time ever heard of EC	Less than a month ago	26	10.5
	Less than six months ago	30	11.9
	Six months to 3 years ago	72	28.8
	Over 3 years ago	121	48.8
Measures to prevent pregnancy after unprotected sex	Yes	231	92.7
	No	18	7.3

*Note:* Data presented as frequency and percentage. ECs: emergency contraceptives

### 3.3 Knowledge of Emergency Contraceptives

**Figure 2:**
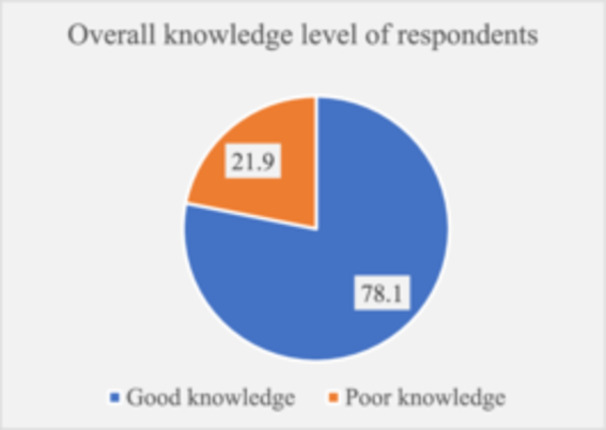
Overall knowledge level of respondents on ECs.

Most of the respondents (79.9%) knew the appropriate time to use emergency contraceptives after unprotected sex. Oral contraceptive pills were known by 61.3% of the students. Almost half of the students were aware that the best time to use oral contraceptive pills (48.9%) and copper Intrauterine devices (44.1%) as emergency contraceptives was within 72 hours of unprotected sex. Again, forty‐three‐point three percent (43.3%) of the students disagree that EC serves as an early method of abortion. More than half of the participants (58.3%) were knowledgeable about the side effects of EC while 49.7% did not know the possible side effects (Table 3). Overall, knowledge of female college students regarding emergency contraception was good at 78.2% (Figure 2).

**Table 3:** Participants' knowledge of ECs.

Variable	Responses	Frequency (*n* = 310)	Percentage (%)
Appropriate time for ECs use	After unprotected sex	199	79.9
	As an ongoing contraceptive	10	4.2
	To abort an unwanted pregnancy	19	7.6
	Don't know	21	8.3
Respondents' knowledge of ECs types	Oral contraceptive pills (OCPs)	153	61.3
	IUCD	32	12.9
	Injectable	44	17.6
	Others	20	8.2
Appropriate time for OCPs	Within 3 days	122	48.9
	3–5 days	47	19
	After a week	7	2.8
	Don't know	73	29.2
Appropriate time for IUD	Within 72 h after unprotected sex	110	44.1
	Within 5 days after unprotected sex	32	12.9
	Even after a week of unprotected sex	10	3.9
	Don't know	98	39.2
ECs as an early method of abortion	Yes	63	25.2
	No	108	43.3
	Don't know	78	31.5
Knowledge of the side effects of ECs	Yes	181	58.4
	No	129	41.6

*Note:* Data presented as frequency and percentage.

Abbreviations: ECs, emergency contraceptives; IUCD, copper intrauterine device; OCPS, oral contraceptive pills.

### 3.4 Attitudes Toward Emergency Contraceptive Practices

**Figure 3:**
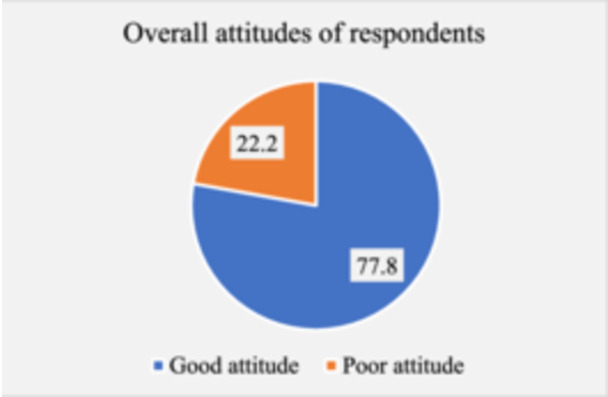
Overall attitude of respondents toward EC use.

Most female college students (94.8%) believed that unwanted pregnancies are problems for young females while 73.5% of them admit that EC service is effective in preventing unwanted pregnancies. More than two‐thirds of students (70.9%) stated they were willing to use EC when the need arises with 70.2% of them highlighting that they will recommend EC for others to use. Moreover, majority (77.3%) of the respondents support the idea of making EC pills accessible and affordable. However, it was indicated by 42.8% of the respondents that they agree it is morally wrong to use EC. Additionally, just below half (45.1%) of the respondents believe ECs encourage promiscuity with 8.3% indicating it is against their religion to practice emergency contraception (Table 4). Overall, the majority of participants (77.8%) had good attitudes toward EC practices whereas 22.2% had poor attitudes (Figure 3).

**Table 4:** Participants' attitudes toward emergency contraceptive practices.

Variable	Responses	Frequency (*n* = 310)	Percentage (%)
Opinion regarding unwanted pregnancy of young females	Yes	294	94.8
	No	16	5.2
Respondents' beliefs in the effectiveness on ECs	Yes	228	73.5
	No	82	26.5
Willingness to use ECs when the need arises	Yes	220	70.9
	No	90	29.1
Recommend to others	Yes	218	70.2
	No	92	29.8
Support idea of making ECs accessible and affordable	Yes	240	77.3
	No	70	22.7
Recommendation for the provision of ECs services	Health worker	190	61.3
	Pharmacists	78	25.2
	Over‐the‐counter medicine seller	37	11.9
	other	5	1.7
Convenience of ECs services	Yes	230	74.1
	No	80	25.9
It is wrong morally to use ECs	Agree	133	42.8
	Neutral	42	13.4
	Disagree	136	43.8
ECs encourage promiscuity	Agree	140	45.1
	Neutral	85	27.5
	Disagree	85	27.5
Unmarried young females can access and use ECs	Agree	220	71.3
	Neutral	37	11.9
	Disagree	52	16.8
Correct usage of ECs is safe	Agree	234	75.6
	Neutral	42	13.4
	Disagree	34	11.1
It is easy and affordable to procure ECs	Agree	177	57.1
	Neutral	61	19.7
	Disagree	72	23.3
ECs is one way of abortion	Agree	92	29.7
	Neutral	48	15.4
	Disagree	170	54.9

*Note:* Data presented as frequency and percentage.

Abbreviation: ECs, emergency contraceptives.

### 3.5 Utilization of Emergency Contraceptives

This study showed that 52.0% of the respondents had used EC before, with majority (87.8%) stating unprotected sex as the main reason for the use of EC. Levonorgestrel‐only pill was identified as the major EC method used by 69.5% of the students. Further, the findings found that 53.6% of the students got their EC commodities from health facilities/healthcare providers. Many of the respondents (71.9%) indicated their willingness to use EC to prevent pregnancy in the event of engaging in unplanned and unprotected sexual affair. It was also highlighted by 72.0% of the respondents that they would recommend EC to a colleague to prevent unplanned pregnancy. About one‐third (36.3%) indicated accessibility/affordability as the major factor influencing the use of EC (Table 5).

**Table 5:** Participants' utilization of emergency contraceptives.

Variables	Responses	**Frequency (*n* = 310)**	**Percentage (%)**
Ever use emergency contraceptive	Yes	161	52.0
	No	149	48.0
Reason for using ECs	Unprotected sex	141	87.8
	Missed pill	17	10.4
	Others	3	1.8
Methods/drugs used	Combined oral pill	41	25.6
	Intrauterine device (IUD)	4	2.4
	Levonorgestrel‐only pill (postinor2)	112	69.5
	Others	4	2.4
Source of used EC commodities	Pharmacists/chemical seller	56	35.0
	Health facility/healthcare providers	86	53.6
	Friends/colleagues	17	10.8
	Others	1	0.6
Willingness to use ECs in the event of unprotected sex	Yes	223	71.9
	No	87	28.1
Willingness to recommend to a friend to prevent unplanned pregnancy	Yes	223	72.0
	No	45	14.5
	Not sure	42	13.5
Reasons for using emergency contraceptives	Accessibility/affordability	113	36.3
	Partner/spousal consent	77	24.7
	Undesirous of pregnancy	59	19.0
	Others	62	20.0

*Note:* Data presented as frequency and percentage.

Abbreviations: ECs, emergency contraceptives; IUD, intrauterine device.

### 3.6 Factors Associated With the Utilization of Emergency Contraceptives

Table 6 illustrates the factors associated with the utilization of emergency contraceptives among female college students. In a univariate logistic regression model, participants from the Midwifery College were found to be 3.06 times more likely to patronize emergency contraceptives than those attending the College of Education [cOR = 3.06, 95% CI (1.90–4.95), *p* < 0.0001]. Similarly, respondents who had good knowledge about emergency contraceptives were 5.53 times more likely to use it compared to those who had poor knowledge [cOR = 5.53, 95% CI (2.89–10.56), *p* < 0.0001]. After adjusting for possible confounders in a multivariable logistic regression model, both institution of training [aOR = 1.96, 95% CI (1.14–3.38), *p* = 0.015] and knowledge [aOR = 4.46, 95% CI (2.28–8.72), *p* < 0.0001] were observed to be significant predictors of emergency contraceptives use (Table 6).

**Table 6:** Sociodemographic characteristics and knowledge associated with emergency contraceptive use among female college students.

Variable	EC utilization^a^		χ (*p*‐value)	cOR (95% CI); *p*‐value	aOR (95% CI); *p*‐value
	No (*n* = 149)	Yes (*n* = 161)			
**Age**					
< 21	22 (7.38)	26 (8.72)	2.3204 (0.509)	Ref	
21–25	103 (34.56)	100 (33.56)		0.82 (0.44–1.54): 0.541	
26–30	16 (5.37)	21 (7.05)		1.11 (0.47–2.63): 0.812	
> 30	3 (1.01)	7 (2.35)		1.97 (0.46–8.56): 0.363	
**Ethnicity**					
Sissala	40 (13.07)	57 (18.63)	2.8698 (0.412)	Ref	
Kasem	10 (3.27)	8 (2.61)		0.56 (0.20–1.55): 0.264	
Dagare	35 (11.44)	32 (10.46)		0.64 (0.34–1.20): 0.165	
Others	62 (20.26)	62 (20.26)		0.70 (0.41–1.20): 0.195	
**Religion**					
Christian	84 (27.45)	97 (31.70)	0.4718 (0.492)	Ref	
Muslim	63 (20.59)	62 (20.26)		0.85 (0.54–1.35): 0.492	
**Institution of training**					
College of education	77 (25.16)	42 (13.73)	21.6697 (< **0.001**)	Ref	Ref
Midwifery college	70 (22.88)	117 (38.2)		3.06 (1.90–4.95): **< 0.0001**	1.96 (1.14–3.38): **0.015**
**Year at college**					
Year 1	44 (14.43)	53 (17.38)	0.4557 (0.796)	Ref	
Year 2	44 (14.43)	48 (15.74)		0.91 (0.51–1.60): 0.734	
Year 3	58 (19.02)	58 (19.02)		0.83 (0.48–1.43): 0.500	
**Knowledge**					
Poor	45 (16.30)	15 (5.43)	30.2334 (< **0.001**)	Ref	Ref
Good	76 (27.54)	140 (50.7)		5.53 (2.89–10.56): **< 0.0001**	4.46 (2.28–8.72): **< 0.0001**

*Note:* Data^a^ presented as frequency (percentage). Bolded *p*‐values are considered statistically significant.

Abbreviations: aOR, adjusted odd ratio; cOR, crude odd ratios; χ, chi‐square value.

## 4 Discussion

Adolescents face a higher risk of complications and death as a result of unplanned pregnancy than older women [19]. The utilization of emergency contraceptives can significantly diminish the likelihood of maternal morbidity and mortality particularly resulting from unsafe abortion procedures. This study investigated the utilization of emergency contraceptives and its associated factors among female college students in rural Ghana. The study revealed that 82.0% of female college students were aware of emergency contraceptives, a finding consistent with similar research conducted in Ghana, which reported an awareness rate of 81% [5]. Similarly, comparable rates were observed in Ethiopia (90.7%) [20], while a lower awareness level of 68% was documented in India [21], 22% in Botswana [22] and 17.3% in Uganda [23]. These findings could be due to the impact of globalization and improved information, communication technologies like media expansion (both mainstream and social media), improved sexual and reproductive health care services advocacy [24].

The major source of information on EC observed in this study was health workers (41.6%) which contradicts several studies where radio/television and colleagues/friends were the major sources of information. Studies conducted by Tesfa et al. [25] and Oseitutu & Ampadu [26] found lower proportions of students obtaining information on EC from health workers (25.2% and 15%) respectively. Other studies reported different primary sources of information on EC. Friends were identified as the main EC information source in a study conducted by Wagner et al. [27], with media also playing a significant role [28]. Misinformation about EC from unreliable sources threatens ongoing efforts to stem unwanted and unintended pregnancies, especially among young women. The promotion and provision of accurate information by health educators in tertiary institutions to improve the safe and effective usage of EC.

Moreover, our research revealed that the majority of participants (78.1%) had a good knowledge level of EC. This is higher than what was reported by Shiferaw et al. (46.8%) among female students in Mizan‐Tepi University, Southwest Ethiopia [29], although consistent with a study conducted in Tamale, Ghana where 86.9% had a good knowledge score [30]. These disparities could be attributed to variations in demographics such as age, educational background, and urban versus rural residency which can influence knowledge levels. The relatively high level of EC knowledge in our study and other Ghanaian study populations suggests that existing educational efforts are having some success. However, there is a need for targeted interventions to ensure that higher knowledge scores translate to improved EC utilization. The implementation of comprehensive sexual and reproductive health education programs by colleges in rural communities could encourage improved EC utilization and practices [31]. Additionally, supporting the implementation of peer education programs where students can educate their peers about EC can effectively improve reproductive health outcomes as students will feel more comfortable with peers compared to older individuals.

The study further found that 48.9% of participants correctly identified the correct time to use EC after unprotected sex (within 72 h), which aligns with published findings of a study conducted in West Ethiopia (49.3%) [17]. However, a sizable proportion (39.2%) did not know the correct effective time for using EC, particularly the IUCD emergency contraception. This finding is lower compared to a study conducted at Nursing and Midwifery Training College Tamale, Northern Region that revealed 54.9% did not know the correct use of IUCD as an emergency contraceptive [30]. This shortcoming might be due to the reliance on friends/colleagues for EC‐related knowledge, the lack of in‐depth knowledge by some health professionals and the lack of counseling centers available on student campuses that handle and promote reproductive and sexual health program services. For students, unplanned pregnancy poses a significant problem, and the management of educational institutions needs to liaise with students in the provision of crucial information and in‐depth knowledge of general contraception methods including ECs practices/utilization. It is particularly concerning because family planning is supposed to be one of the major contents of the curriculum for the midwifery programmed. Improved EC knowledge will aid in the development of their ability to provide effective and accurate EC data to prevent unplanned or unwanted pregnancies. The efficacy of emergency contraceptives requires correct timing of usage. The longer one waits to use emergency contraception after an unprotected sexual encounter, the more likely it is that an unwanted pregnancy will occur [32]. Knowledge of emergency contraceptives is hence vital in preventing or reducing unwanted pregnancy incidences among students which could adversely impact their academic prospects [32]. These challenges highlight the importance of broadening and including detailed information on general contraceptives, particularly EC, in the educational curriculum.

Moreover, this current study revealed that 70.9% of respondents expressed a willingness to use emergency contraceptives (EC) if needed, while 70.2% indicated they would recommend EC to others. These findings align with previous studies, which reported that 88.2% [33] and 71.2% [34] of participants were willing to use EC and recommend it to colleagues under similar circumstances. Overall, participants' attitudes towards emergency contraceptives were good at 77.8%. This is consistent with research documented by the Health Science and Medical students of Arba Minch, Ethiopia who revealed an overall positive attitude towards EC as 88.2% [35]. It is disturbing to find that about 45.1% believed EC usage encourages sexual promiscuity and 43.8% agreeing it is morally wrong to use EC. This contradicts the findings of other studies that revealed the majority of the respondents disagreeing that it is morally wrong to use EC and 43% not agreeing it promotes sexual promiscuity [36, 37]. However, our findings are consistent with those of Mohammed et al.'s study in Ghana, which reported that 69.8% of participants believed emergency contraceptives promote indiscriminate sexual behavior [30]. In many African societies, including Ghana, premarital sex and non‐procreative sex can be stigmatized particularly in rural areas due to traditional beliefs emphasizing sexual purity and procreation within marriage [31]. The existing literature does not back the notion that using EC encourages sexual promiscuity or discourages the usage of other contraceptive approaches. These findings are concerning, considering that the participants were college students, often regarded as agents of change within their communities. This is particularly significant for student health professionals, such as midwives, who will soon graduate and provide healthcare services, including family planning and contraceptive counseling. This may influence their attitude toward providing accurate information, education, counseling and appropriate care to females seeking to patronize EC and possibly other contraceptive means. Our results emphasize the importance of stepping up advocacy and education campaigns aimed at demystifying negative attitudes toward EC and other methods of contraception.

The current study showed that 52.0% of the respondents had ever used EC of which 69.5% used postinor‐2, 25.6% used combined oral pills, and 2.4% used intrauterine contraceptive devices. These rates are significantly higher than those reported in a study conducted at Takoradi Polytechnic in Ghana, where only 28.4% of participants indicated prior use of emergency contraception [38]. This finding is lower than a study in Ethiopia, where nearly 83.0% of participants reported utilizing emergency contracetives [39], but aligns with a study conducted at Arbaminch University in Ethiopia, which documented a utilization rate of 58.8% [35]. These differences could be related to the high proportion of sexually active students which is similar to the later Ethiopian study. In addition, the study identified availability/affordability and partner consent as reasons for the use of EC. This is consistent with a US‐based study that indicated ease of obtaining ECs commodities and moral acceptability as predictors of emergency contraceptive use [40]. Public health intervention by the Ghana health service should integrate comprehensive EC education into sexual and reproductive health services, as well as consider subsidizing EC costs, expanding distribution, and training healthcare providers to offer non‐judgmental counseling in rural communities and institutions [41].

The factors associated with the utilization of emergency contraceptives were assessed in this study. Findings revealed that the institution being attended and the knowledge level of respondents were significantly associated with the usage of emergency contraceptives. Participants attending the Midwifery College were more likely to utilize emergency contraceptives compared to those attending the College of Education. This could be because family planning is a significant component of the curricula for the midwifery programs. After a multivariate binary logistic regression, female college students with good knowledge were 4.6 times more likely to have better utilization of emergency contraceptives than those with poor knowledge. The capacity to understand and deliver accurate and useful information about EC to prevent unintended or undesired pregnancies increases as their expertise in EC grows through formal learning in school. The possibility of respondents from the Midwifery College and other maternal training centers to have good knowledge with regard to EC types, appropriate time of usage and its adverse effect can have a significant influence on their patronage of EC commodities compared to their counterparts. Moreover, our research corroborates with another study that identified higher knowledge to be associated with contraceptive usage [42]. On the other hand, it contradicts a study conducted in Ghana that found no significant association between higher knowledge and the use of contraception [43]. Similar research conducted in Spain [44] and the United States of America [45] discovered that participants who had obtained formal education and EC‐related content had a considerably higher knowledge of EC.

While this study provides novel insights into the factors associated with emergency contraceptive (EC) utilization among female college students in rural Ghana, it was not without limitations. The cross‐sectional study design approach does not allow for the establishment of causality in the factors influencing EC utilization. Recall bias could have affected participants' ability to recall experiences and perspectives regarding EC utilization, knowledge, and attributes. Additionally, social desirability bias might have made respondents underreport behaviors due to stigma, discrimination, and social rejection.

## 5 Conclusion

Despite good knowledge and awareness, proper use of EC is still suboptimal due tothe lack of understanding and misperception. The institution attended and knowledge level were significant predictors of emergency contraception utilization. Particular educational programs and curriculum upgrades are recommended to policymakers and educators in rural communities to increase EC use and prevent unintended pregnancies in female undergraduates. These targeted educational initiatives and advocacy would help debunk myths and misconceptions and encourage EC utilization.

## Author Contributions

**Muhusin Alhassan:** conceptualization, investigation, writing – original draft, writing – review & editing, methodology. **Yaa Nyarko Adjeso:** conceptualization, writing – review and editing. **Clement Tiimim Yanbom:** formal analysis, writing – review and editing, data curation. **Samuel Kwame Sopuruchi Agomuo:** writing – original draft, writing – review and editing, formal analysis, data curation.

## Conflicts of Interest

The authors declare no conflicts of interest.

## Transparency statement

The lead author Samuel Kwame Sopuruchi Agomuo affirms that this manuscript is an honest, accurate, and transparent account of the study being reported; that no important aspects of the study have been omitted; and that any discrepancies from the study as planned (and, if relevant, registered) have been explained.

## Data Availability

The data that support the findings of this study are available from the corresponding author upon reasonable request.
